# The New Year

**Published:** 1887-01

**Authors:** 


### HALL'S JOURNAL OF HEALTH

TRUTH DEMANDS NO SACRIFICE; ERROR CAN MAKE NONE.

VOL. 34. JANUARY, 1887. No. 1.

### THE NEW YEAR.

We wish our patrons, each and all, a happy new year. When, twelve months ago, the fortunes of the JOURNAL were committed to the present management, the question whether or not the liberal policy upon which it then entered, and has since maintained, would be acceptable to all or even a majority of its readers, hung in doubt, for it was scarcely to be expected that so radical a change from its previous conservatism would meet with unanimous concurrence, but so far as we are advised, such, indeed, has been the case. Not only have its old time patrons stood manfully by it, but there has been added a greater harvest of new ones, than in any previous year, not excepting its most flourishing days. This is a good sign in a double aspect, for it shows that editor and reader are in sympathy with each other in respect to a knowledge of those living ideas which it is the policy of the JOURNAL to present, not, however, with the expectation that every reader will coincide with the more radical of them, but that even those who take opposite ground, lay too much value upon the diverse opinions of others to wish to remain in ignorance of them, knowing, too, that agitation broadens the pathway that leads ever upward. This is an age of progress, not only in respect to material things, but of the spiritual also, for there comes a tardy recognition that all that is of value to man—all that he gains in knowledge and experience—even those wonderful discoveries and inventions by means of whereof he is able to command the subtile forces of nature, and bend them to his will, are the outcome of an undying principle within him acting and being acted upon through the wonderful mechanism of his cerebral apparatus, of which it no more forms a part, than steam forms a part of the mechanism of the locomotive engine, to which it gives force and motion. It is earnestly to be hoped that the time is only a little way off when the recognition of this inestimable truth shall become general, and that through the awakening of all that is highest and noblest within them, all men may be induced to walk uprightly, doing only good, with no dread of the present and no fear of the unknown future. When if ever that time comes, the world will be peopled with wiser and more humane generations.

It is our purpose to continue, as heretofore, to lay before our readers such useful information as we are able to gather, upon all subjects suited to the family and the fireside, for *ours* is strictly a home publication, and *health* in its more ample signification, comprehends the moral and intellectual, no less than the physical state of being; hence, whatever conduces to the profit of either is suited to our columns.

The past year has demonstrated that we have no patrons so weak or so bigotted as to reject the truth out of fear that its acceptance will have a tendency to overturn preconceived ideas. Falsehood, however gilded and draped, and templed, and domed, even though it has descended to us from remote centuries, wearing the face of a martyr, and the sword of a victor, must surely give way before the all-conquering march of human progress. No matter how long and how devoutly an untruth has been cherished, age only adds to its deformity in contrast with the ever-increasing knowledge attendant upon the researches of the learned. To-day, science and philosophy are no longer in opposition, but with a firm reliance upon the divine center of being, they have been brought to a recognition of their kinship, and walk side by side amid the harmonies of nature and the sublime record of the ages, tracable through unnumbered centuries from the primal rock to the fruited tree fashioned for the sustenance of man. More than all, the unreasoning acceptance of error, is spiritual stagnation, which indisposes to investigation and is fatal to progress.

"Truth demands no sacrifice; errors can make none."

Such is the maxim which we have placed at the head of the JOURNAL. Nothing that is *not true* can be availabe for good, and error is not only valuless but hurtful. The more we have of it, the worse we are off. In dealing with any question or philosophy it will be our aim to make no claim for it that will not bear the strictest investigation, nor shall we shrink from its introduction to our columns simply because of its unpopularity. There was a time, not so very far distant, when to deny that our earth was the center of the universe, was so unpopular as to endanger life, because it amounted to a denial of a thelogical dogma. Then no publication could have resisted this stupid error and survive. Fortunately, it is not so now.

Again wishing our readers great happiness and prosperity throughout the year whose beginning is to-day, and with the assurance that we shall hereafter spare no pains and omit no duty which will subserve their interest or add to their enjoyment, in our monthly visits to their several homes, we commend the JOURNAL to their favor and indulgence.

